# High mortality rate of obstetric critically ill women in Rwanda and its predictability

**DOI:** 10.1186/s12884-021-03882-7

**Published:** 2021-05-25

**Authors:** Alcade Rudakemwa, Amyl Lucille Cassidy, Théogène Twagirumugabe

**Affiliations:** 1 Ruhengeri Referral Hospital , North Province Ruhengeri, Rwanda; 2 Department of Anesthesiology, Wake Forest University School of Medicine, North Carolina Winston-Salem, USA; 3 College of Medicine and Health Sciences, University of Rwanda, University Teaching Hospital of Butare, Butare, Rwanda

**Keywords:** Obstetrics, Intensive care unit, Critical care, Maternal mortality prediction

## Abstract

**Background:**

Reasons for admission to intensive care units (ICUs) for obstetric patients vary from one setting to another. Outcomes from ICU and prediction models are not well explored in Rwanda owing to lack of appropriate scores. This study aimed to assess reasons for admission and accuracy of prediction models for mortality of obstetric patients admitted to ICUs of two public tertiary hospitals in Rwanda.

**Methods:**

We prospectively collected data from all obstetric patients admitted to the ICUs of the two public tertiary hospitals in Rwanda from March 2017 to February 2018 to identify reasons for admission, demographic and clinical characteristics, outcome including death and its predictability by both the Modified Early Obstetric Warning Score (MEOWS) and quick Sequential Organ Failure Assessment (qSOFA). We analysed the accuracy of mortality prediction models by MEOWS or qSOFA by using logistic regression adjusting for factors associated with mortality. Area under the Receiver Operating characteristic (AUROC) curves is used to show the predicting capacity for each individual tool.

**Results:**

Obstetric patients (*n* = 94) represented 12.8 % of all 747 ICU admissions which is 1.8 % of all 4.999 admitted women for pregnancy or labor. Sepsis (*n* = 30; 31.9 %) and obstetric haemorrhage (*n* = 24; 25.5 %) were the two commonest reasons for ICU admission. Overall ICU mortality for obstetric patients was 54.3 % (*n* = 51) with average length of stay of 6.6 ± 7.525 days. MEOWS score was an independent predictor of mortality (adjusted (a)OR 1.25; 95 % CI 1.07–1.46) and so was qSOFA score (aOR 2.81; 95 % CI 1.25–6.30) with an adjusted AUROC of 0.773 (95 % CI 0.67–0.88) and 0.764 (95 % CI 0.65–0.87), indicating fair accuracy for ICU mortality prediction in these settings of both MEOWS and qSOFA scores.

**Conclusions:**

Sepsis and obstetric haemorrhage were the commonest reasons for obstetric admissions to ICU in Rwanda. MEOWS and qSOFA scores could accurately predict ICU mortality of obstetric patients in resource-limited settings, but larger studies are needed before a recommendation for their use in routine practice in similar settings.

## Background

Intensive care unit (ICU) admissions of obstetric patients are infrequent and little is known about incidence and outcome in low resources countries. Global incidence for obstetric patients varies from 0.4 to 16.0 % of all ICU admissions [1]. In high and middle income countries, 0.13–0.76 % of all births results in maternal ICU admission, whereas in sub-Saharan Africa (sSA) the rates are 0.24–0.95 %. However, only 0.65–1.5 % of ICU admissions in high and middle income countries are obstetric patients compared to 1.25–6.7 % in sSA [2–5].

Various reasons for admission of obstetric patients to ICUs have been identified and the prevalence of each admitting diagnosis varies between countries. Hypertensive disorders and obstetric hemorrhage are highly prevalent in high and middle income countries, whereas the most common reasons for admission in low-income countries in Africa are hemorrhage and sepsis [2, 3, 5, 6].

Mortality among obstetric patients admitted to ICU remains obviously higher in low-income countries compared with high-income countries. While this ICU mortality was estimated at 3.5 % in Netherlands, it was almost 10 times higher in Kenya and South Africa [5, 7, 8]. Predicting maternal mortality, however, remains challenging as currently available ICU severity scores are not suitable for obstetric patients admitted to ICUs such as CIPHER (Collaborative Integrated Pregnancy High-dependency Estimate of Risk) and ICNARC (Intensive Care National Audit and Research Centre). These showed high discrimination, but carry many challenges in low resource settings with limited laboratory capacities [9, 10]. Evidence showed that the Modified Early Obstetric Warning Score (MEOWS) developed by the Confidential Enquiry into Maternal and Child Health (CEMACH) and the quick Sequential Organ Failure Assessment (qSOFA) are useful in early detection of physiological derangements [11–13]. There is no specific publication on obstetric admissions to ICU and evidence that these tools may predict outcome for obstetric patients admitted to ICU in Rwanda. Therefore, this study was conducted to determine the main reasons for ICU admission for obstetric patients, outcomes of obstetric patients admitted to ICU and to evaluate accuracy of MEOWS and qSOFA to predict mortality for obstetric patients admitted to ICU in public referral hospitals in Rwanda.

## Methods

The study was conducted in the two university teaching hospitals in Rwanda: Centre Hospitalier Universitaire de Butare (CHUB) and Centre Hospitalier Universitaire de Kigali (CHUK) which are tertiary hospitals with a capacity of 454 beds, including 6 beds for ICU and 526 beds of which 7 are dedicated for ICU. We conducted a prospective cross-sectional study for all obstetric patients admitted from March 2017 to February 2018.

We included all women who were admitted to ICU with ongoing pregnancies or within 42 days after childbirth or termination of pregnancy. Patients were followed from admission to discharge from ICU. ICU-trained nurses collected data comprising demographics such as age, parity, vital signs, reason for admission recorded from ICU files, ICU interventions including inotropic agents or vasopressors needs, administration of blood components, surgical procedures, mechanical ventilation and renal replacement therapy. ICU length of stay (LOS) in days and outcome at discharge from ICU (dead or alive) were also recorded. ICU LOS was calculated as the number of days spent in ICU with zero days for a stay > 24 h. Vital signs at admission were used to calculate MEOWS and qSOFA scores accordingly.

Data were analysed using Statistical Package for Social Sciences (IBM SPSS Statistics for Windows, version 22.0. Armonk, NY: IBM Corp). Descriptive results are reported as percentages, mean +/- standard deviation and median with interquartile range (IQR). Incidence of obstetric admission to ICU was calculated based on numbers of obstetric patients admitted in ICU over numbers of all births in both hospitals during the study period. Rate of obstetric admission to ICU was based on the total number of all ICU admissions. Comparisons of frequencies or median scores between survivors and non-survivors within ICU were made by using Chi-square or Mann-Whitney U tests. *P*-value > 0.05 was considered as statistically significant. Variables with significant or a trend of association with outcome (*p* < 0.25) were included in a logistic regression model to identify independent predictors of mortality in models assessing accuracy of MEOWS or qSOFA. Generated adjusted Receiver Operating Characteristic Curves (ROC) and adjusted areas under the ROC (aAUROC) are presented. Moreover, AUROCs with 95 % Confidence Intervals for each individual risk score as MEOWS or qSOFA (unadjusted analysis) are also calculated and curves provided for comparison with the adjusted ones.

## Results

### Demographic data and severity scores of obstetric patients on admission to ICUs

During the study period, 747 women were admitted to ICUs of either CHUB or CHUK. Of them, 94 (12.8 %) were admitted during pregnancy or within 42 days after birth or termination of pregnancy. Overall, 4,999 women were admitted for pregnancy or labor in the two facilities, corresponding to 1.8 % of obstetric patients admitted to ICU.

Mean age of obstetric patients was 29.8+/-6.5 years and 52 of them (55.3 %) were admitted in their first or second pregnancy (Table 1). Seventy patients (74.5 %) were admitted after birth, 13 (13.8 %) post-abortion or following complications of ectopic pregnancies, while eleven (11.7 %) were pregnant at the time of admission to ICU. Of 70 patients admitted after birth, 44 (62.9 %) gave birth by cesarean section and 26 (37.1 %) had vaginal birth (Table 1).

**Table 1:** Characteristics of obstetric patients admitted in ICU in our study

Variables	Range	Frequency	Mean ± SD
**Age (in years)**			29.82 ± 6.507
**Parity at admission to ICU**	1–2	52(55.3 )	
	≥ 3	42 (44.7 )	
**Period of admission**	During pregnancy	11 (11.7)	
	Post-abortion/ectopic pregnancy	13 (13.8)	
	Post-partum	70 (74.5)	
**Admitted in post-partum period ** (*n* = 70)			
** Mode of delivery**	Cesarean section	44 (62.9)	
	Normal delivery	26 (37.1)	

### Reasons for admission to ICU and interventions during ICU stay

The most common reasons for admission to ICU for obstetric patients in the two public tertiary hospitals was sepsis (*n* = 30; 31.9 %), followed by obstetric hemorrhage (*n* = 24; 25.5 %), while other conditions including cardiomyopathy, stroke, embolism and trauma were diagnosed in 19 women (20.2 %). Hypertensive disorders of pregnancy (*n* = 16; 17 %) and malaria (*n* = 5; 5.3 %) were the least represented (Table 2).

**Table 2:** Reasons for admissions and interventions performed on study patients during their ICU stay

Variables		Number of patients	Percentage (%)
**Reason for ICU admissions**			
Hemorrhage		24	25.5
Hypertensive disorders of pregnancy		16	17.0
Sepsis		30	31.9
Malaria		5	5.3
**Others**		**19**	**20.2**
Cardiomyopathy		5	5.3
Embolism		7	7.5
Trauma		5	5.3
Stroke		2	2.1
**Interventions done**			
Invasive mechanical ventilation		90	95.7
Blood transfusion		33	35.1
Inotropics/vasopressors support		47	50.0
Re-operation		5	5.3
Hemodialysis		4	4.3

Mechanical ventilation was the most frequently performed intervention in 95.7 % (*n* = 90), inotropic agents and/or vasopressors were needed in 50.0 % (*n* = 47), blood components in 35.1 % (*n* = 33), surgery during ICU stay was performed on 5.3 % (*n* = 5) while hemodialysis was done in 4.3 % (*n* = 4) (Table 2). Some patients received more than one intervention as dictated by clinical conditions and/or severity of disease (Table 2).

Average length of ICU stay was 6.6 ± 7.525 days and 51 (54.3 %) study participants died in ICU.

**Fig. 1:**
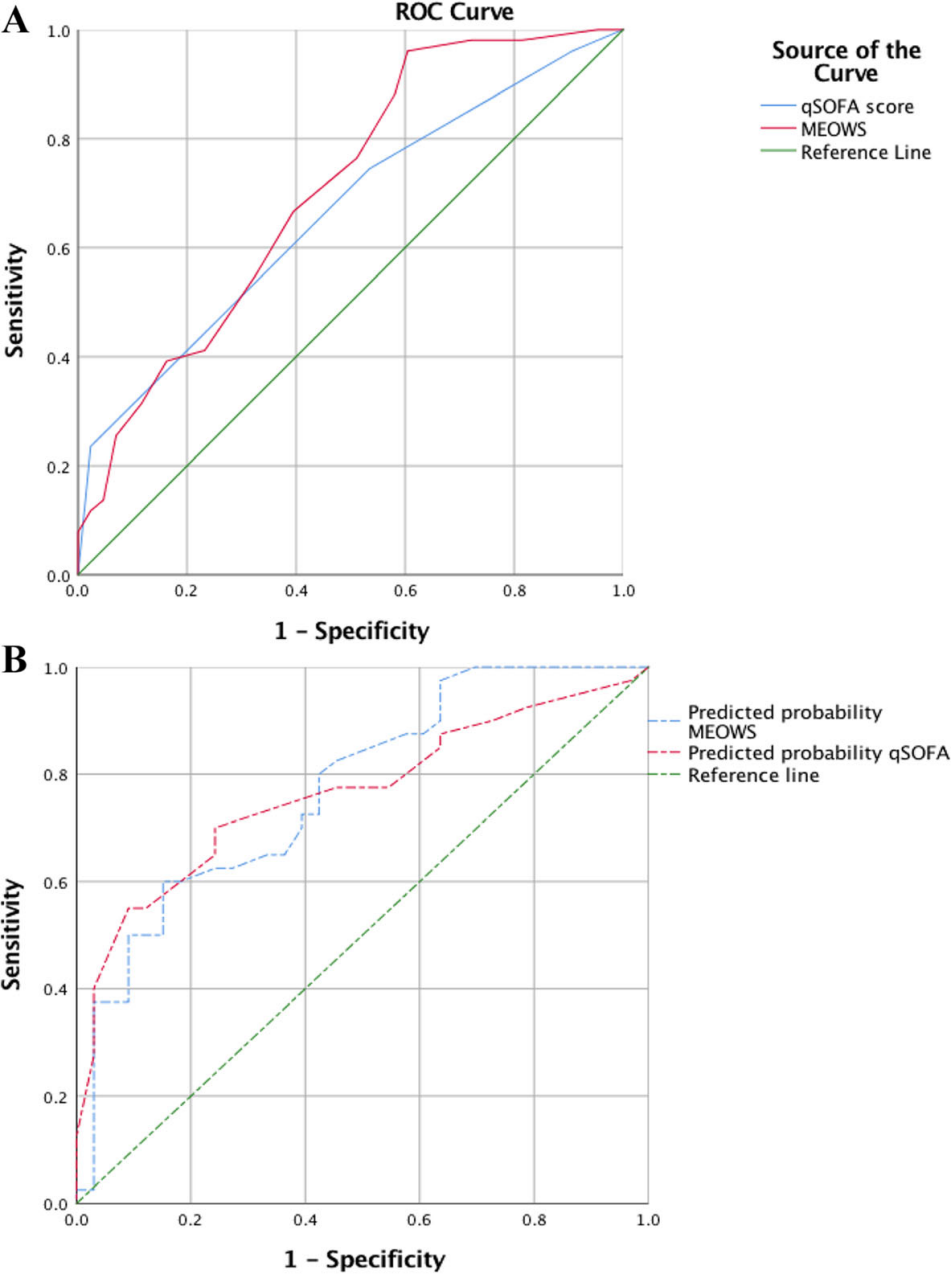
**a**: Unadjusted AUROC for prediction of ICU mortality by qSOFA and MEOWS. **b**: Adjusted AUROC for prediction of ICU mortality by qSOFA and MEOWS

Analysis of factors associated with ICU survival showed that neither setting nor both mode of birth and the reason for admission were associated with poorer outcome (Table 3). There was, however, a statistically significant difference of MEOWS scores between survivors and non-survivors. Median MEOWS was 7 (IQR: 6; 8) for survivors compared with 8 (IQR: 6; 12) for non-survivors (*p* = 0.001). Also median qSOFA score was 2 (IQR: 1; 2) in the two groups. Multivariable logistic regression, adjusting for reason of admission and cesarean section before admission showed that MEOWS was an independent predictor for ICU mortality (aOR 1.25; 95 % CI 1.07–1.46) and one point increase of qSOFA-score was independently associated with increased odds of ICU mortality by 181 % (aOR 2.81; 95 % CI 1.25–6.30) (Table 4). In the analysis of both mortality prediction models, aAUROCs are presented in Fig. 1b. The aAUROC for MEOWS and qSOFA scores were 0.773 (95 % CI 0.666–0.880) and 0.764 (95 % CI 0.654–0.873), showing a fair discrimination capacity for mortality prediction by the two models. Besides that, unadjusted AUROCs for individual MEOWS and qSOFA scores are also presented (Fig. 1a). Unadjusted AUROC for qSOFA and for WEOWS were 0.662 (95 % CI 0.553–0.771) and 0.705 (95 % CI 0.600-0.811).

**Table 3:** Factors associated with survival rate

Variable		*N* = 94	Survivors n (%)	Non-survivors n (%)	*p*-value
**Setting**	CHUK	60	25 (26.6)	35 (37.2)	0.389
	CHUB	34	18 (19.1)	16 (17.0)	
**Reason for admission**	Hemorrhage	24	11 (11.7)	13 (13.8)	0.078
	Sepsis	30	9 (9.6)	21 (22.3)	
	Others	40	23 (24.5)	17 (18.1)	
**Mode of delivery**	C-section	44	24 (25.5)	20 (21.3)	0.058
	Vaginal	26	9 (9.6)	17 (18.1)	
**MEOWS [Median (IQR)]**			7 (6;8)	8(6;12)	0.001
**qSOFA [Median (IQR)]**			2 (1;2)	2(1;2)	0.008

**Table 4:** Multivariable Logistic regression for MEOWS and qSOFA/predictors of ICU mortality

Variables		Adjusted OR [95 %CI]	*p*-value
**MEOWS**		1.25[1.07–1.46]	0.005
Caesarean		0.39[0.12–1.22]	0.106
Reason for admission			
Haemorrhage		1 (Ref)	
Sepsis		1.72[0.42–6.94]	0.449
Others		0.65[0.18–2.39]	0.517
**qSOFA**		2.81[1.25–6.30]	0.012
Caesarean		0.33[0.11–1.02]	0.054
Reason for admission			
Haemorrhage		1 (Ref)	
Sepsis		1.50[0.38–5.93]	0.559
Others		0.88[0.24–3.31]	0.855

## Discussion

Obstetric admissions to the ICU in public referral hospitals in Rwanda represent 12.8 % of all ICU admissions and are likely to happen for 1.8 % of all births in these hospitals. These rates are relatively high compared with those from high income countries, ranging between 0.22 and 0.76 % [2, 14, 15]. Our findings remain comparable to those prevailing in some middle-income countries such as Brazil (1.29 %) and Turkey (1 %) [16, 17]. Moreover, these are similar to those described in a study from Nigeria which showed that obstetric patients represented 17.3 % of all ICU admissions and 2.1 % of all births [18].

Rates of obstetric admissions in ICU of sSA hospitals may even be relatively higher than reported. This could be linked to limited ICU capacity as the number of ICU beds in Rwanda represents only around 1.5 % of all hospital beds, whereas this number varies between 2 and 9 % in majority of high income countries [19, 20]. This challenge of scarcity of ICU beds is actually shared with other sSA countries, even if some countries may report rates between 0.24 and 0.97 % [5, 8, 21].

The severity of conditions for obstetric patients in our study, as evidenced by the proportions of those who needed ventilator support (90 %) and inotropic agents or vasopressors (50 %) is another argument for the number of obstetric admissions in ICUs in Rwanda to be higher when capacity would allow. The two leading causes of obstetric admission to ICU in Rwanda were sepsis (31.9 %) and obstetric haemorrhagic shock (25.5 %). These conditions substantially differ in incidence from those prevailing in high income countries. Therefore, these may also partly explain discrepancies in ICU mortality given that sepsis and septic shock generally incur high mortality both in high income countries like the United States and in low income countries such as Rwanda [22, 23]. With respect of hemorrhagic shock, potential coagulation disorders resulting from delays to achieve hemostasis, lack of readily available blood products and massive transfusion may also explain high mortality in our setting.

ICU mortality was as high as 53.4 %. Relatively worse outcome has been seen in other sSA countries like Burkina Faso where this reached 60 % [24]. Poor outcome of such patients may be attributable to limited management capacity of our ICUs and delays in which patients were finally admitted in severe condition. This poor outcome is shared with other ICU patients in Rwanda where mortality, among all ICU admissions, culminated in 48.7 % in 2016 [23]. High rates of sepsis and associated mortality for obstetric patients in Rwandan settings have also been reported from one of the teaching hospitals [25].

Numerous tools for mortality prediction have been developed for general ICU patients such as the Acute Physiology and Chronic Health Evaluation (APACHE), the Simplified Acute Physiology Score (SAPS) and the Sequential Organ Failure Assessment (SOFA). Their generalization to obstetric patients remains challenging [26]. Our study evaluated the accuracy of easy tools such as MEOWS and qSOFA for prediction of ICU mortality in obstetric patients. The models have shown rather good discriminative capacities with fair AUROC for both models. These findings corroborate others from a study in Australia on patients suspected with sepsis, showing that a positive qSOFA (≥ 2 points) identified patients at higher risk of in-hospital mortality or longer ICU stay [27]. Good discriminative power of qSOFA for mortality prediction was also reported in a study from India about septic patients in ICU or non-ICU wards [28]. Findings on MEOWS are comparable with those from a study in the United Kingdom, where MEOWS had high sensitivity and good specificity to early detect morbidity among obstetric patients outside ICU [11]. Thus, MEOWS can also be applied as a simple bedside model for the purpose of predicting mortality. This, however, needs further exploration in ICU patients from various settings.

### Strengths and limitations

 Data for this study were prospectively collected from two tertiary hospitals that have relatively well equipped and staffed ICUs than other public hospitals. Therefore, majority of obstetric patients with critical conditions are admitted in those hospitals. This may give findings from the study the strength to be generalizable to obstetric ICU patients in Rwanda. The study, however, also has some limitations including small sample size. It could have been necessary to collect data for a longer period owing to the limited number of ICU beds in the country [29]. Moreover, patients were followed just during their ICU stay and outcome after discharge from ICU is another limitation. Despite that, results from this study are fundamental for further exploration to find affordable and accurate mortality prediction tools for obstetric patients admitted in ICUs of resource-limited settings.

## Conclusions

Obstetric hemorrhagic shock, sepsis and septic shock are major reasons for obstetric admissions to ICUs in Rwanda. A relatively very high ICU mortality was observed among obstetric patients. MEOWS or qSOFA scores for ICU mortality prediction in those patients perform fairly well. Further studies with larger sample size are, however, needed before generalized use of these tools for mortality prediction in critically ill obstetric patients.

## Data Availability

Datasets are available from the corresponding author on reasonable request.

## Abbreviations

aOR: Adjusted Odd ratio
APACHE: Acute, Physiology, Age and Chronic Health Evaluation
AUROC: Area Under the Receiver Operating Characteristic Curve
aAUROC: Adjusted Area Under the Receiver Operating Characteristic Curve
CEMACH: Confidential Enquiry into Maternal and Child Health
CHUB: Centre Hospitalier Universitaire de Butare
CHUK: Centre Hospitalier Universitaire de Kigali
CI: Confidence Interval
Corp.: Corporate
IBM: International Business Machines
ICU: Intensive Care Unit
IQR: Interquartile range
MEOWS: Modified Early Obstetric Warning Score
NY: New York
OR: Odd ratio
*p*-value: Calculated Probability
qSOFA: Quick Sequential Organ Failure Assessment
ROC: Receiver Operating Characteristic curve
SAPS: Simplified Acute Physiology Score
SCCM: Society of Critical Care Medicine
SD: Standard deviation
SOFA: Sequential Organ Failure Assessment
SPSS: Statistical Package for Social Sciences
sSA: Sub-Saharan Africa(n)
